# The Scandinavian ACL registries 2004–2007: baseline epidemiology

**DOI:** 10.3109/17453670903350107

**Published:** 2009-10-01

**Authors:** Lars-Petter Granan, Magnus Forssblad, Martin Lind, Lars Engebretsen

**Affiliations:** ^1^Oslo Sports Trauma Research Center, Norwegian School of Sport Sciences, Ullevål StadionOsloNorway; ^2^National Knee Ligament RegistryBergenNorway; ^3^Capio Artro Clinic AB and Stockholm Sports Trauma Research CenterSophiahemmet, StockholmSweden; ^4^Division of Sports Trauma, Orthopaedics Department, Aarhus University HospitalAarhusDenmark; ^5^Orthopaedics Center, Ullevaal University Hospital and Faculty of Medicine, University of OsloNorway

## Abstract

**Background and purpose** No prospective surveillance systems have been available for monitoring the outcome of cruciate ligament surgery in Scandinavia (Denmark, Norway, and Sweden). In the present paper we describe the Scandinavian ACL registries including their main function, similarities, and preliminary baseline results.

**Methods** The Scandinavian registries were established in 2004 (Norway) and 2005 (Denmark and Sweden). The Danish and Swedish registries were originally based on the Norwegian registry, and there is no overriding difference between the three. In Denmark, all hospitals and clinics are legally bound to report to an approved national database. In Norway and Sweden, the registries are based on voluntarily reporting by surgeons.

**Results** The annual incidence of primary ACL reconstructions is higher in Denmark than in Norway, except in females younger than 20 years. Among Scandinavian surgeons, there is a similar approach to the patients. Differences do, however, exist regarding choice of grafts, choice of implants, and choice of treatment of simultaneous meniscal and cartilage injuries; the proportion of ACL reconstructions performed as outpatient surgery; and the use of prophylactic anticoagulation. Clinically, the preoperative KOOS scores are not significantly different between the Scandinavian registries, except that Denmark reports more symptoms both pre- and postoperatively.

**Interpretation** The Scandinavian national ACL registries will generate new data about ACL reconstructions. They will contribute important knowledge regarding ACL epidemiology. They will be the only source of data on the performance of a wide range of different implants and techniques. In addition, they will hopefully have an impact on the selection of methods for ACL reconstructions in Scandinavia and elsewhere.

## Introduction

Over the last two decades, Scandinavian national arthroplasty registries have generated important knowledge and have served as an important quality control tool. Until Norway started the world's first national knee ligament registry in 2004, there had been no prospective national surveillance systems to monitor the outcome of knee ligament surgery (Granan et al. 2008).

We describe the 3 Scandinavian—Danish, Norwegian, and Swedish—knee ligament registries, their main function, and their similarities. Furthermore, preliminary baseline results for primary ACL reconstructions are presented from the start of the registries until late 2007.

## Patients and methods

The Scandinavian registries were established in June 2004 (Norway), January 2005 (Sweden), and July 2005 (Denmark). The latter two were based on the Norwegian registry. There is no overriding difference between these registries. Details of the Norwegian ACL registry have been described previously by Granan et al. (2008).

The Norwegian and Swedish registries depend on surgeons reporting on a voluntary basis to the registries. In Denmark, a law passed in June 2006 made it compulsory for all public and private hospitals and clinics to report to the approved national, clinical databases. Reporting to the databases in Denmark and Sweden is organized through a secure internet portal, thus minimizing the costs of daily running. In Norway, the registry relies on paper-based reporting, mainly due to the close cooperation with the Norwegian Arthroplasty Register (NAR), which makes use of an identical system.

In Denmark, 90% of all orthopedic departments have been contributing to the registry, with an average compliance of 85% of the primary ACL reconstructions performed. In Norway, all hospitals performing ACL surgeries have contributed, with a total compliance of 97%. In Sweden, some of the smaller hospitals with small volumes of ACL surgery have not been included in the registry, yet more than 71% of the hospitals have contributed to the registry.

Follow-up with KOOS (Knee injury and Osteoarthritis Outcome Score) forms is carried out by all 3 registries. In Denmark, these follow-ups are done at 1, 5, and 10 years postoperatively. In Norway they are done at 2, 5, and 10 years postoperatively, and in Sweden they are done at 1, 2, 5, and 10 years postoperatively.

All registries provide annual reports, both on a national basis and for the individual hospitals. Sweden also offers an online database where clinics can analyze their own statistics at any time. The Danish database is managed by a special university center that manages all Danish national orthopedic databases. In Norway, the technical responsibility rests with Helse Vest IKT AS, which manages all Norwegian national orthopedic databases. In Sweden, the Capio Artro Clinic in Stockholm is responsible for the registry on a daily basis.

For the present study, data regarding common and comparable variables related to the primary ACL reconstruction were extracted (hospital, sex, age at injury and surgery, activities causing injury, time to surgery, frequency of cartilage and meniscal injuries, meniscal resections, and cartilage treatments, choice of graft, choice of graft fixation devices, duration of surgery, prophylactic antibiotics and anticoagulation, outpatient surgery, number of reconstructions, and preoperative and postoperative KOOS).

### Ethics

In Norway the participation is voluntary, and all patients are asked to sign an informed consent form before surgery. The consent form contains information about the Norwegian ACL registry, the type of information that is recorded, data protection, and the procedure for follow-ups, and also informs the patient that he or she may be invited to participate in research projects at a later stage. The registration forms are signed by the surgeons but they cannot be traced in the registry database since the surgeon's identity is not recorded, due to a mutual agreement between the Norwegian orthopedic registries. For follow-ups, the patients are identified by their unique social security number (including date of birth), which is assigned to all residents of Norway. The Norwegian ACL registry has been approved by the Norwegian Data Inspectorate. In Denmark and Sweden, no consent is necessary for national clinical databases. In Denmark and Sweden, the social security number is used to identify patients in the ACL registries and crosscheck data with national healthcare registries.

## Results

The total number of primary ACL reconstructions reported was 4,972 in Denmark, 5,329 in Norway, and 7,331 in Sweden. The percentage of male patients was 57% in Norway, 58% in Sweden, and 60% in Denmark. Of the Danish patients, 1,939 (39%) had simultaneous meniscal injuries and 825 (17%) had cartilage injuries. For the Norwegian patients the corresponding figures were 2,914 (55%) and 1,456 (27%), and in Sweden they were 2,536 (35%) and 2,001 (27%). The median age of the patients at the time of injury varied between 23 years (Sweden) and 27 years (Denmark), while the median age at the time of surgery varied between 25 years (Sweden) and 30 years (Denmark). The median time (in months) from injury to surgery varied between 7 (Norway) and 10 (Sweden). The median duration of surgery varied between 68 min (Denmark) and 71 min (Sweden). Outpatient surgery was performed in 38% of the cases in Norway, 56% of the cases in Sweden, and 79% in Denmark. In all countries, 99% of the patients received prophylactic antibiotics while the use of prophylactic anticoagulation varied between 17% in Denmark and 78% in Norway. These surgeries were conducted in 37 hospitals in Denmark, 52 hospitals in Sweden, and 60 hospitals in Norway. Hamstring autografts were the most frequently used graft in all of Scandinavia (61% in Norway, 71% in Denmark, and 86% in Sweden). Soccer was the most frequent cause of injury (Norway 40%, Sweden 41%, and Denmark 50%) (Table 1).

**Table 1.** Variables in the registration forms reported to the Scandinavian ACL registries

Characteristics	Denmark	Norway	Sweden
Primary ACL reconstructions			
total number	4,972	5,329	7,331
annual average^b^	1,886	1,520	2,444
Hospitals	37	60	52
Age at surgery, median (range)	30 (10–71)	27 (12–67)	25 (8–67)
Age at injury, median (range)	27 (7–70)	25 (6–65)	23 (5–66)
Males	60%	57%	58%
Grafts:			
hamstring	71%	61%	86%
BPTB	22%	38%	14%
other	7%	< 1%	< 1%
Meniscal injuries			
total	1,939 (39%)	2,914 (55%)	2,536 (35%)
resection	1,591 (79%)	2,002 (69%)	2,007 (80%)
Cartilage injuries			
total	825 (17%)	1,456 (27%)	2,001 (27%)
treatment	482 (55%)	293 (20%)	401 (20%)
Duration of surgery^a^, median (range), min	68 (30–210)	70 (10–240)	71 (14–330)
Time to surgery, median (range), months	9 (0–371)	7 (0–482)	10 (0–527)
Outpatient surgery	79%	38%	56%
Prophylactic antibiotics	99%	99%	99%
Prophylactic anticoagulation	17%	78%	41%
Activities that most frequently caused injury	Soccer 50%	Soccer 40%	Soccer 41%
	Team handball 20%	Team handball 15%	Downhill skiing^c^ 13%
	Downhill skiing^c^ 14%	Downhill skiing^c^ 13%	Floor ball 8%
na: data not available.				^a^ Skin-to-skin time for isolated primary ACL reconstructions.				^b^ This average is lower than expected due to inclusion of the very first months of running time of the individual registries.				^c^Alpine skiing, telemark skiing, and snowboarding.			

Clinically, the KOOS data showed no significant national differences in any of the subscales, either preoperatively or postoperatively, except for poorer symptom scores in the Danish patients (Table 2). The Danish KOOS data are based on 50% of the patients in the registry, while the Norwegian data are based on 88% of the registered patient population.

**Table 2.** Preoperative and follow-up KOOS scores in the Scandinavian ACL registries

Time point/Subscale	Denmark	Norway	Sweden
Preoperatively			
Pain	72	78	76
Symptoms	57	75	70
Function in ADL	79	88	85
Function in sport and recreation	40	40	43
Knee-related QOL	40	31	33
1 year post-operatively			
Pain	84	na	85
Symptoms	61	na	78
Function in ADL	90	na	92
Function in sport and recreation	63	na	64
Knee-related QOL	60	na	60
2 years post-operatively			
Pain	na	89	86
Symptoms	na	86	80
Function in ADL	na	97	92
Function in sport and recreation	na	70	66
Knee-related QOL	na	69	62
na: data not available.			

The annual incidence of primary ACL reconstructions in Norway was 34 per 100,000 inhabitants (Granan et al. 2008), while in Denmark the incidence was 38 per 100,000 (Lind et al. 2009), and in Sweden 32 per 100,000 (Table 3). On the other hand, the real population at risk—that is, the 16–39-year age group—had an incidence of 85 primary ACL reconstructions per 100,000 inhabitants in Norway (Granan et al. 2008), while the Danish incidence was 91 per 100,000 for the 15–39-year age group (Lind et al. 2009) and the Swedish incidence was 71 per 100,000 for the 20–39-year age group.

**Table 3.** The annual incidence of primary ACL reconstructions per 100,000 citizens in Scandinavia

Age	Females			Males		
	Denmark	Norway	Sweden	Denmark	Norway	Sweden
10–19	71	76	88	71	47	59
20–29	85	64	62	191	112	117
30–39	79	42	39	137	77	65
40–49	52	24	27	69	38	31
50–59	10	8	6	15	5	5
60–69	3	0.5	0.2	2	1	0.4

## Discussion

In general, the registries provided detailed epidemiological data. Based on conservative estimates, the Scandinavian ACL registries are expected to generate an annual average of 2,500 patients in each of the Danish and Swedish registries and 1,600 patients in the Norwegian registry. After 5 years more than 30,000 cases will be in the registries, yielding data such as revision rates, KOOS, and outcome relating to the various techniques and implants used.

It is also important to emphasize what the registries will not be able to show. There is no radiographic follow-up of the ACL reconstructed patients. Consequently, data regarding the development of radiographically verified osteoarthritis will not be obtainable. The choice of not doing radiographic follow-ups is due to both financial restraints and the intention not to put additional demands on the hospitals that are beyond their own follow-up routines. More advanced investigations (e.g. gait analysis and muscle strength) are also omitted due to the same constraints.

It is the main intention of the registries to contribute to quality control and improvement of the surgical cruciate ligament procedures. This may be done through establishing evidence-based national guidelines and protocols for surgical procedures and rehabilitation. To understand the importance of reported failures, we need to know the actual number of reconstruction and revision surgeries that are performed. Nordic arthroplasty registries have previously provided accurate data of sufficient quality. The Norwegian ACL registry has calculated that if 14 patients—registered in the database—with one specific fixation device show failure, this may be considered a failure of that specific device (Granan et al. 2008). This will enable the registries to give early warnings on poor procedures and devices, and to identify prognostic factors etc.

The registries must provide information for the orthopedic community at regular intervals on the outcomes of surgical treatment of the cruciate ligaments with different methods. The hard endpoints are clear and unequivocal, i.e. revision reconstruction and total knee replacement. Causality of failure may not be sufficiently and accurately documented in the registries, but it will provide information as to where there may be potential problems and direct future analysis and studies toward these areas. Since the registries will provide real-time information and can thus be analyzed on an ongoing basis, they have the potential to reveal problems long before they would be reported by traditional methods (e.g. RCTs). This will undoubtedly benefit all interested parties, not least the patients.

An important limitation in these registries is bias due to limitation in follow-ups. From the Norwegian registry, we know that baseline compliance is high both with respect to registration forms (97%) and KOOS forms (88%). Mandatory reporting has been instituted in Denmark. This might be the most important condition to obtain a high and sustainable compliance. Due to the unique Scandinavian social security numbers, it is easy to reach every patient and thus increase the response rate in the follow-ups.

There are still issues for which the Scandinavian registries have no solutions. Currently, due to logistic and diagnostic issues, patients who do not receive surgical treatment for their ACL injury are not included in the Scandinavian registries. Thus, no data on the outcome of nonoperatively treated ACL injuries can be obtained. Another limitation in these registries is the use of revision as a primary endpoint. This is suboptimal, since an unknown number of patients accept to live with an inferior clinical outcome to avoid more surgery. However, if they undergo surgery for debridement or arthroscopic surgery for other indications, they will be detected in the registry. Knee arthroplasty has limitations as an endpoint because it can take several decades before a patient with a poorly functioning knee is accepted as a knee arthroplasty candidate. Not all patients with ACL insufficiencies develop osteoarthritis to a degree where knee arthroplasty is indicated (Lohmander et al. 2007).

The registration of potential risk factors other than type of surgical procedure may be subject to selection bias. The data items recorded are a minimal set suited for a paper-based or web-based reporting system, designed not to exceed one page. As such there has to be a careful, continuously updated selection of what would be expected to be the most important risk factors. Thus, there is no way of knowing the influence of the variables that are omitted. Finally, there might be limitations due to differences between Scandinavia and other countries regarding indications for surgery and patient success criteria.

Prospective national registries have several advantages. Inclusion of cases from an entire nation generates a high volume of data. This in turn will lead to the possibility of drawing early conclusions. Another advantage is due to the nature of cohort studies: ongoing accumulation of short-term and long-term follow-up data. Finally, there is the advantage of monitoring development, implementation, and evolution of new—and old—techniques, implants, prophylactic medications, and so forth. Although RCTs are the gold standard in research methods and are immensely valuable for detailed testing, they are insufficient when assessing techniques. An RCT aimed at demonstrating a 5% difference in revision rates after ACL surgery would need nearly 500 patients in each group, which is far more than is usually included in a typical RCT in knee ligament surgery.

It is possible to develop ACL registries that are entirely web-based—as demonstrated by the Danish and Swedish registries—and that are accessible and cost effective. Some constraints exist due to the national legislation and infrastructure of different countries. Ultimately, an emerging international cooperation is expected to increase quality, to remove barriers, and to create an open-minded international discussion about methods and results in primary ACL reconstructions.

The different annual numbers in the Scandinavian registries are due to the differences in population sizes. Even though Norway is the smallest country, it has the largest number of hospitals. This is probably due to a scattered population in a relatively long and narrow country.

The data in Table 1 reflect the similar approach of the Scandinavian surgeons to patients. Some national variations do, however, exist: surgeons in Sweden and Denmark prefer hamstring grafts to a much greater extent than surgeons in Norway. The reporting and classification of cartilage injuries in Norway was inconsistent in the early years. This might explain why surgeons in Norway report more than 50% as many cartilage injuries as their colleagues in Denmark, and surgically treat less cartilage injuries than surgeons in Denmark. However, the Swedish data are identical to the Norwegian data. On the other hand, Norway reports substantially more meniscal injuries than Denmark and Sweden, but treats relatively fewer injuries. This probably reflects national attitudes. The variation in ACL reconstructions performed as outpatient surgery probably reflects the variation in the structure of healthcare systems. The large variation in the use of prophylactic anticoagulation is also of interest, but postoperative thrombo-embolic complications are not recorded.

Regarding choice of autograft and fixation, in more than two-thirds of cases the number of different implants used varied between 1 and 3 in the different registries (data not shown). This gives an overall total of 4–6 different implants when looking at various grafts and their different fixation sites. This variation in the Scandinavian countries might be due to personal preferences, to the skills of medical company sales teams, or to local financial decisions—or most likely a combination of these factors. Clinically, there were no significant differences in any of the KOOS subscales, either pre- or postoperatively, between the Scandinavian countries. The only exception found was that Danish patients had clinically significantly poorer symptom scores than their Norwegian and Swedish counterparts, both preoperatively and postoperatively. Furthermore, the Danish and Swedish baseline KOOS data revealed an unsatisfactory compliance rate, for unknown reasons. The baseline KOOS data (Table 2) constitute the most comprehensive dataset published to date, and should be regarded as the reference values for preoperative KOOS in ACL injured patients.

There are as yet no explanations for the large discrepancies between the Scandinavian incidence data (Table 3). These differences should be investigated more thoroughly in separate studies.

The Scandinavian national ACL registries will generate new data about ACL reconstructions and they will contribute to a better understanding of ACL epidemiology. They will be the only source of data on performance of a wide range of different implants and techniques. They will also influence the selection of methods for ACL reconstructions in Scandinavia and hopefully in other countries in the future.
